# Correction: Mediation and moderation effects of health system structure and process on the quality of mental health services in Ghana–structural equation modelling

**DOI:** 10.1371/journal.pone.0246137

**Published:** 2021-01-22

**Authors:** Eric Badu, Anthony Paul O’Brien, Rebecca Mitchell, Akwasi Osei

There are errors in Fig 1 and Fig 2 regarding the covary between observed variable “G” (under latent variable Structure) and observed variable “M” (under latent variable Process). In Fig 2, the covary for the modification of the model is performed between observed variables under the same latent variable. Goodness of Fit: RMSEA = 0.05; 90% CI, lower bound = 0.050; upper bound = 0.063; pclose = 0.062 Probability RMSEA < = 0.05; Comparative fit index (CFI) = 0.940; Tucker-Lewis index (TLI) = 0.917; Coefficient of determination = 0.88. Please see the correct Fig 1 and Fig 2 here.

**Fig 1:**
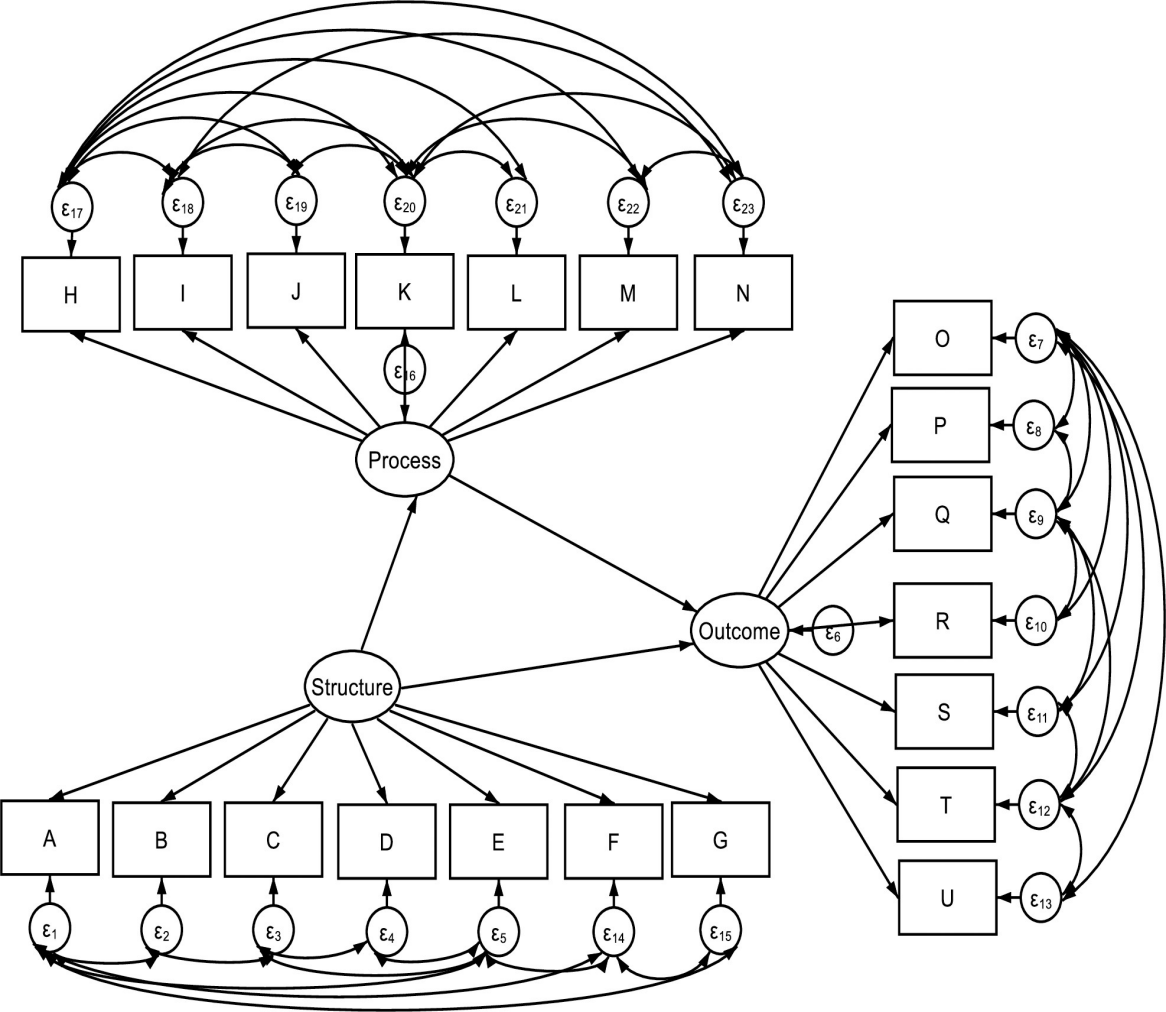
Hypothesised structural equation model.

**Fig 2:**
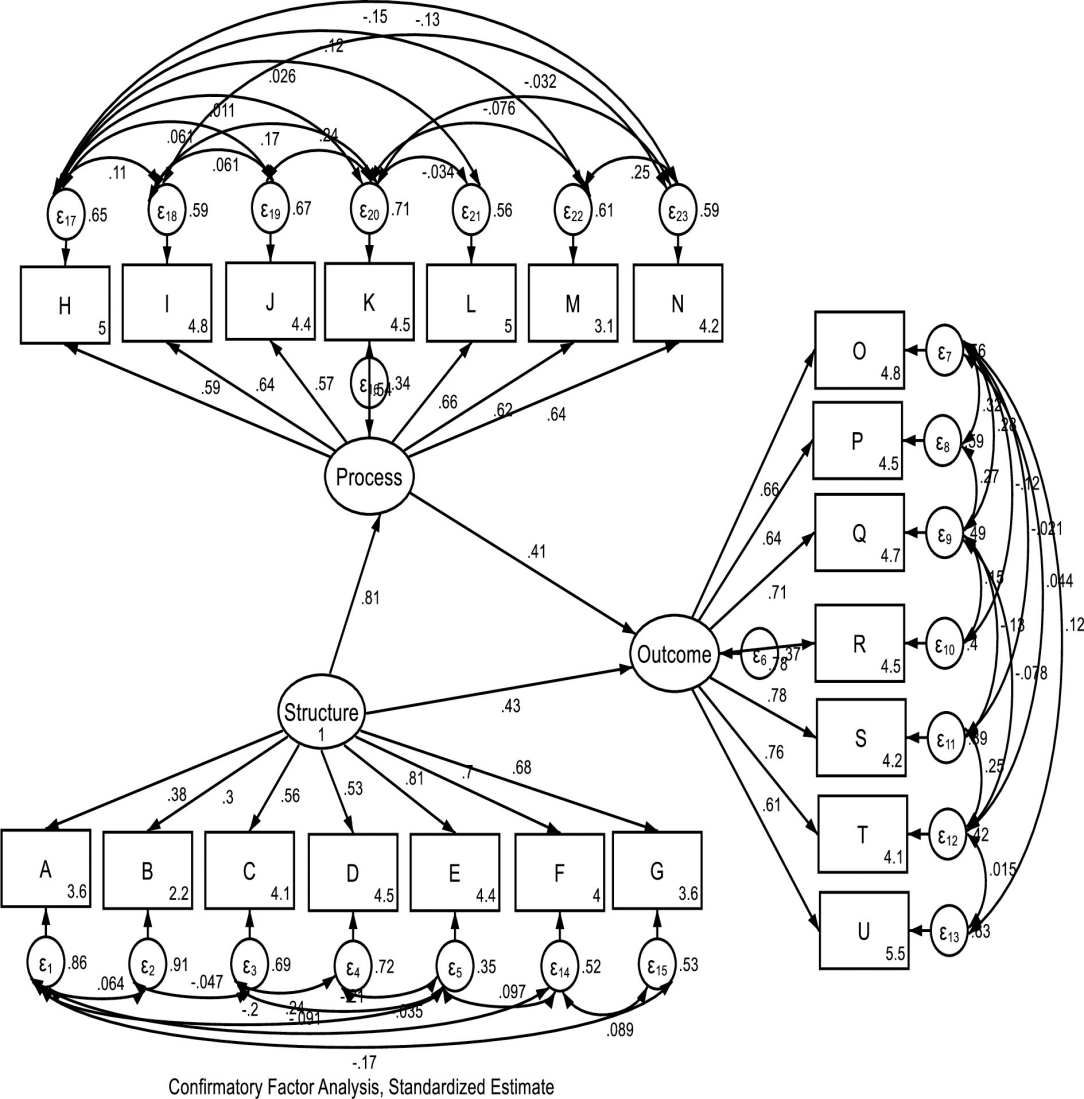
Standardized coefficient estimates of hypothesized SEM.

These errors also occur in the Confirmatory factor analysis results subsection of the Results and in Table 4. The correct paragraph is:

As shown in Table 3, the correlation matrix demonstrates sufficient convergent and discriminant correlation between the VSS-54 construct and WHODAS construct. Fig 1, graphically describes the hypothesized SEM. Results from the CFA showed that the hypothesized model had a good fit with the Residual Mean Square of Approximation (RMSEA) = **0.05**; 90% CI, lower bound = **0.050**; upper bound = **0.063**; pclose = **0.062**; Comparative fit index (CFI) = **0.940**; Tucker-Lewis index (TLI) = **0.917**; Coefficient of determination = **0.88**. Details of the standardized coefficient estimates for CFA is represented in Fig 2 and Table 4. The path model result shows that the health system *structure* was significantly related to the *process* and *outcome* (β = **0.47**; p<0.001). Similarly, the health systems *structure* construct was mediated by the *process* construct in its relationship with the *outcome* (β = 0.346; p<0.001).

Please see the correct Table 4 here.

**Table 4:** Standardized coefficient estimates for CFA.

Variables	Latent constructs	β	SE	95% CI
*Structural*				
**Process**	Outcome	0.47^**^	**0.13**	**0.21–0.73**
**Structure**	Outcome	0.60^**^	**0.17**	**0.27–96**
**Structure**	Process	1.01^**^	**0.16**	**0.69–1.32**
*Measurement*				
**Accessibility**	Structure	**1.00**		
**Affordability**	Structure	0.96^**^	**0.19**	**0.58–1.34**
**Managing Side effects**	Structure	1.38^**^	**0.22**	**95–1.81**
**Response of Service to Crises**	Structure	1.19^**^	**0.19**	**0.80–1.57**
**Listen to the worries of Relatives**	Structure	1.89^**^	**0.27**	**1.36–2.42**
**Recommendation to relatives**	Structure	1.73^**^	**0.25**	**1.22–2.21**
**Information to Relatives**	Structure	1.89^**^	**0.27**	**1.32–2.41**
**Competency of psychiatrist**	Process	**1.00**		
**Psychiatrist listen & understand illness**	Process	1.15^**^	**0.11**	**93–1.37**
**Instructions about appointment**	Process	1.07^**^	**0.11**	**0.85–1.30**
**Cooperation between service providers**	Process	.96^**^	**0.11**	**0.73–1.19**
**Confidentiality and respect for your right**	Process	1.16^**^	**0.11**	**0.93–1.38**
**Information about diagnosis and prognosis**	Process	1.52^**^	**0.17**	**1.18–1.85**
**Explanation of procedures and approaches**	Process	1.14^**^	**0.13**	**0.97–1.50**
**Attaining wellbeing**	Outcome	**1.00**		
**Knowledge & understanding**	Outcome	0.01^**^	**0.07**	**0.85–1.16**
**Symptoms**	Outcome	1.08^**^	**0.08**	**0.92–1.16**
**Self-care**	Outcome	1.25^**^	**0.10**	**1.05–1.46**
**Relationship**	Outcome	1.31^**^	**0.10**	**1.10–1.52**
**Work/vocational skills**	Outcome	1.31^**^	**0.10**	**1.10–1.52**
**Satisfaction**	Outcome	0.79^**^	**0.07**	**0.65–0.93**

**Significance p<0.01; Goodness of Fit: RMSEA = 0.05; 90% CI, lower bound = 0.050; upper bound = 0.063; pclose = 0.062 Probability RMSEA < = 0.05; Comparative fit index (CFI) = 0.940; Tucker-Lewis index (TLI) = 0.917; Coefficient of determination = 0.88
