# Supplementary material for: Mediation and moderation effects of health system structure and process on the quality of mental health services in Ghana – structural equation modelling
Source: PLoS One. 2020 May 22;15(5):e0233351. doi: 10.1371/journal.pone.0233351 (PMC7244180; doi:10.1371/journal.pone.0233351)
Supplement: S1 Survey — (PDF) [file pone.0233351.s001.pdf]

# The Quality of Mental Health Services in Ghana: Providers and Consumers Perspectives

| | |
|---|---|
| Record ID | __________________________________ |
| Interviewer ID | __________________________________<br>(Interviewer) |
| Name of Health Facility | ◯ Accra Psychiatric Hospital<br>◯ Pantang Psychiatric Hospital<br>◯ Ankafu Psychiatric Hospital<br>◯ KATH<br>◯ BA Regional Hospital<br>◯ Other<br>(Name of health facility) |
| SD1. What is the mental health service the consumer is receiving? | ◯ Outpatient service (visiting review)<br>◯ Discharged from In-patient (0 - 12 months)<br>(Type of service) |
| SD2. What did you do when you first experienced this condition? | ◯ Visited the prayer centre/church<br>◯ Visited herbalist<br>◯ Visited the imam/spiritual centre<br>◯ Visited the health facility<br>(Treatment pathways) |
| SD3. If you visited health facility, what was the type of service provider? | ◯ Health centre<br>◯ Clinic<br>◯ Hospital<br>◯ Psychiatric hospital<br>(Type of health service provider) |
| SD4. Who introduced you to this point of receiving mental health service? | ◯ General practitioner (medical doctor, nurses)<br>◯ Community Mental health worker<br>◯ Pastor at prayer centre<br>◯ Family member<br>◯ Friends, colleague or community member<br>(Person who introduced you to the mental health service) |
| SD5. Age | __________________________________<br>(Age) |
| SD6. Gender | ◯ Male<br>◯ Female<br>(Gender) |
| SD7. Education | ◯ None<br>◯ Basic (Primary and JHS)<br>◯ Senior High School/Vocational/Technical<br>◯ Tertiary<br>(Education) |

| | |
|---|---|
| SD8. Marital Status | ◯ Single<br>◯ Married<br>◯ Co-habitation<br>◯ Separated<br>◯ Widow<br>(Marital Status) |
| SD10. Primary Occupation | ◯ Student<br>◯ Unemployed<br>◯ Skilled (eg, teaching, banking, nurse)<br>◯ Semi (eg, artisan, mechanics, seamstress)<br>◯ Unskilled (eg, farmers, drivers, food venders)<br>(Occupation) |
| SD11. Religion | ◯ Christianity<br>◯ Islamic<br>◯ Traditional<br>◯ Other<br>(Religion) |
| SD12. NHIS Status | ◯ Insured (active)<br>◯ Uninsured<br>(Insurance) |
| SD13. What is your current location? | ◯ Urban<br>◯ Peri-Urban<br>◯ Rural<br>(Location) |

**Let talk about your satisfaction with the behaviour and skills of professionals. Please choose the answer that appears most appropriate. If you are unsure about which response to give to a question, the first response you think of is often the best one. I will read out each question to you, along with the five response options (1= Terrible, 2 = Mostly dissatisfied, 3= Mixed, 4 Mostly satisfied and 5 Excellent).**

**What is your overall feeling about the following:**

| | Terrible | Mostly dissatisfied | Mixed | Mostly satisfied | Excellent | Not applicable |
|---|---|---|---|---|---|---|
| PB1. The professional knowledge and competence of psychiatrists? | ○ | ○ | ○ | ○ | ○ | ○ |
| PB2. The professional knowledge and competence of psychologists? | ○ | ○ | ○ | ○ | ○ | ○ |
| PB3. The thoroughness of psychiatrists? | ○ | ○ | ○ | ○ | ○ | ○ |
| PB4. The thoroughness of psychologists? | ○ | ○ | ○ | ○ | ○ | ○ |
| PB5. The professional knowledge and competence of nurses? | ○ | ○ | ○ | ○ | ○ | ○ |
| PB6. The professional knowledge and competence of social workers? | ○ | ○ | ○ | ○ | ○ | ○ |
| PB7. The thoroughness of nurses? | ○ | ○ | ○ | ○ | ○ | ○ |
| PB8. The thoroughness of social workers? | ○ | ○ | ○ | ○ | ○ | ○ |
| PB9. The personal manner of psychiatrists? | ○ | ○ | ○ | ○ | ○ | ○ |
| PB10. The personal manner of psychologists? | ○ | ○ | ○ | ○ | ○ | ○ |
| PB11. The ability of psychiatrists to listen to and understand your problems? | ○ | ○ | ○ | ○ | ○ | ○ |
| PB12. The ability of psychologists to listen to and understand your problems? | ○ | ○ | ○ | ○ | ○ | ○ |
| PB13. The personal manners of nurses? | ○ | ○ | ○ | ○ | ○ | ○ |
| PB14. The personal manners of social workers? | ○ | ○ | ○ | ○ | ○ | ○ |

| | | | | | | |
|---|---|---|---|---|---|---|
| PB15. The nurses' knowledge about you and your medical history? | ○ | ○ | ○ | ○ | ○ | ○ |
| PB16. The ability of nurses to listen to and understand your problems? | ○ | ○ | ○ | ○ | ○ | ○ |
| PB17. The ability of social workers to listen to and understand your problems? | ○ | ○ | ○ | ○ | ○ | ○ |
| PB18. The behaviour and manners of reception or secretarial staff on the telephone or when you meet them? | ○ | ○ | ○ | ○ | ○ | ○ |
| PB19. The instructions about what to do on your own between appointments; the clarity, practicality etc. of recommendations? | ○ | ○ | ○ | ○ | ○ | ○ |
| PB20. The cooperation between service providers (if you are treated by more than one professional)? | ○ | ○ | ○ | ○ | ○ | ○ |
| PB21. The appropriateness of referrals to your GP or other specialist if needed? | ○ | ○ | ○ | ○ | ○ | ○ |
| PB22. The continuity of care (seeing the same staff) you have received? | ○ | ○ | ○ | ○ | ○ | ○ |
| PB23. The confidentiality and respect for your rights? | ○ | ○ | ○ | ○ | ○ | ○ |
| PB24. The punctuality of the professionals when you come for an appointment? | ○ | ○ | ○ | ○ | ○ | ○ |

**Let continue to talk about the information you received when receiving mental health services in the last year. I will read out each question to you, along with the five response options (1= Terrible, 2 = Mostly dissatisfied, 3= Mixed, 4 Mostly satisfied and 5 Excellent). Please choose the answer that appears most appropriate.   If you are unsure about which response to give to a question, the first response you think of is often the best one.**

**What is your overall feeling about the following:**

| | Terrible | Mostly dissatisfied | Mixed | Mostly satisfied | Excellent | Not applicable |
|---|---|---|---|---|---|---|
| IN1. The explanations of specific procedures or approaches used? | ○ | ○ | ○ | ○ | ○ | ○ |
| IN2. How information was given to you about your problem (diagnosis) and what to expect (prognosis)? | ○ | ○ | ○ | ○ | ○ | ○ |
| IN3. The publicity or information about available mental health services? | ○ | ○ | ○ | ○ | ○ | ○ |

**Let talk about issues on accessibility of the mental health services you have received in the last year. I will read out each question to you, along with the five response options (1= Terrible, 2 = Mostly dissatisfied, 3= Mixed, 4 Mostly satisfied and 5 Excellent). Please choose the answer that appears most appropriate. If you are unsure about which response to give to a question, the first response you think of is often the best one.**

**What is your overall feeling about the following:**

| | Terrible | Mostly dissatisfied | Mixed | Mostly satisfied | Excellent | Not applicable |
|---|---|---|---|---|---|---|
| AC1. The appearance, comfort level and physical layout of the facilities (e.g. the waiting rooms and the offices)? | ○ | ○ | ○ | ○ | ○ | ○ |
| AC2. The cost of the service to you (e.g. prescription charges)? | ○ | ○ | ○ | ○ | ○ | ○ |

**Let talk about your perception regarding the efficacy of mental health services you received from the facility. I will read out each question to you, along with the five response options (1= Terrible, 2 = Mostly dissatisfied, 3= Mixed, 4 Mostly satisfied and 5 Excellent). Please choose the answer that appears most appropriate. If you are unsure about which response to give to a question, the first response you think of is often the best one.**

**What is your overall feeling about the following:**

| | Terrible | Mostly dissatisfied | Mixed | Mostly satisfied | Excellent | Not applicable |
|---|---|---|---|---|---|---|
| EF1. The effectiveness of services in helping you to attain wellbeing and preventing relapse? | ○ | ○ | ○ | ○ | ○ | ○ |
| EF2. The effect of services in helping you deal with your problems? | ○ | ○ | ○ | ○ | ○ | ○ |
| EF3. The effectiveness of the service in helping you to improve your knowledge and understanding of your problems? | ○ | ○ | ○ | ○ | ○ | ○ |
| EF4. The effect of services in helping to relieve symptoms? | ○ | ○ | ○ | ○ | ○ | ○ |
| EF5. The effectiveness of the service in improving the relationship between you and your closest relative? | ○ | ○ | ○ | ○ | ○ | ○ |
| EF6. The effectiveness of the service in helping you to improve your self-care (e.g. take care of your personal hygiene, your diet, your room)? | ○ | ○ | ○ | ○ | ○ | ○ |
| EF7. The effectiveness of the service in helping you establish good relationships with people outside your family (e.g. friends, neighbours, colleagues at work, etc.)? | ○ | ○ | ○ | ○ | ○ | ○ |
| EF8. The effectiveness of the service in helping you to improve your ability to work? | ○ | ○ | ○ | ○ | ○ | ○ |

**Let talk about your perception regarding the involvement of your relatives in the mental health services you have received in the last year. I will read out each question to you, along with the five response options (1= Terrible, 2 = Mostly dissatisfied, 3= Mixed, 4 Mostly satisfied and 5 Excellent). Please choose the answer that appears most appropriate. If you are unsure about which response to give to a question, the first response you think of is often the best one.**

**What is your overall feeling about the following:**

| | Terrible | Mostly dissatisfied | Mixed | Mostly satisfied | Excellent | Not applicable |
|---|---|---|---|---|---|---|
| R1. The ability of psychiatrists to listen to and understand the worries your main carer (relative or friend) may have about you? | ◯ | ◯ | ◯ | ◯ | ◯ | ◯ |
| R2. The ability of psychologists to listen to and understand the worries your main carer (relative or friend) may have about you? | ◯ | ◯ | ◯ | ◯ | ◯ | ◯ |
| R3. The acommendations made to your closest relative about how they could help you? | ◯ | ◯ | ◯ | ◯ | ◯ | ◯ |
| R4. How information was given to your main carer (relative or friend) about your problem (diagnosis) and what to expect (prognosis)? | ◯ | ◯ | ◯ | ◯ | ◯ | ◯ |
| R5. The effectiveness of the service in helping your main carer (relative or friend) deal better with your problems? | ◯ | ◯ | ◯ | ◯ | ◯ | ◯ |
| R6. The effectiveness of the service in helping your main carer (relative or friend) improve their understanding of your problems? | ◯ | ◯ | ◯ | ◯ | ◯ | ◯ |

**Let talk about the type of mental health interventions you have received in the last year. I will read out each question to you, along with the five response options (1= Terrible, 2 = Mostly dissatisfied, 3= Mixed, 4 Mostly satisfied and 5 Excellent). Please choose the answer that appears most appropriate. If you are unsure about which response to give to a question, the first response you think of is often the best one.**

**What is your overall feeling about the following:**

| | Terrible | Mostly dissatisfied | Mixed | Mostly satisfied | Excellent | Not applicable |
|---|---|---|---|---|---|---|
| SE1. The response of the service to crises or urgent needs during office hours? | ◯ | ◯ | ◯ | ◯ | ◯ | ◯ |
| SE2. The arrangements made for after-hours emergencies (nights, weakened and public holidays)? | ◯ | ◯ | ◯ | ◯ | ◯ | ◯ |
| SE3. The help you have received for side effects from medications (if occurred)? | ◯ | ◯ | ◯ | ◯ | ◯ | ◯ |

---

SE4. In the last year, have you been prescribed medication?

◯ Yes
◯ No
(Receive prescription for medication)

---

SE4a. If you answered YES, please what is your overall feeling about this/them?

◯ Terrible
◯ Mostly dissatisfied
◯ Mixed
◯ Mostly satisfied
◯ Excellent
(If you have received prescription for medication)

---

SE4b. If you answered NO, Do you think you would have liked to receive this/them?

◯ No
◯ Don't know
◯ Yes
(If you have not received prescription for medication)

---

SE5. In the last year, did you receive help from mental health professionals to improve your capacity to cope with your social and working life (e.g. going to public offices, doing housework, getting on with your family and others)?

◯ Yes
◯ No
(Receive help to cope with social and working life)

---

SE5a. If you answered YES, what is your overall feeling about this/them?

◯ Terrible
◯ Mostly dissatisfied
◯ Mixed
◯ Mostly satisfied
◯ Excellent
(If you have received help for social and work life)

| | |
|---|---|
| SE5b. If you answered NO, do you think you would have liked to receive this/them? | ◯ No<br>◯ Don't know<br>◯ Yes<br>(If you have not received help for social and work life) |
| SE6. In the last year, did you have the opportunity to meet alone, on a regular basis, with your Therapist (e.g. in order to help you understand your problems and/or change your behaviour in some way)? | ◯ Yes<br>◯ No<br>(Meeting therapist regularly) |
| SE6a. If you answered YES, please what is your overall feeling about this/them? | ◯ Terrible<br>◯ Mostly dissatisfied<br>◯ Mixed<br>◯ Mostly satisfied<br>◯ Excellent<br>(If you meet therapist regularly) |
| SE6b. If you answered NO, please do you think you would have liked to receive this/them? | ◯ No<br>◯ Don't know<br>◯ Yes<br>(If you do not meet therapist regularly) |
| SE7. In the last year, did you have compulsory treatment in a psychiatric hospital? | ◯ Yes<br>◯ No<br>(Have compulsory treatment) |
| SE7a. If you answered YES, please what is your overall feeling about this/them? | ◯ Terrible<br>◯ Mostly dissatisfied<br>◯ Mixed<br>◯ Mostly satisfied<br>◯ Excellent<br>(If you received compulsory treatment) |
| SE7b. If you answered NO, please do you think you would have liked to receive this/them? | ◯ No<br>◯ Don't know<br>◯ Yes<br>(If you have received compulsory treatment) |
| SE8. In the last year, did you have meetings with your family and therapist (eg occupational therapist, psychologist, art therapist) with the aim of improving/changing the relationships between family members? | ◯ Yes<br>◯ No<br>(Meeting family and therapist) |
| SE8a. If you answered YES, please what is your overall feeling about this/them? | ◯ Terrible<br>◯ Mostly dissatisfied<br>◯ Mixed<br>◯ Mostly satisfied<br>◯ Excellent<br>(If you have meetings with family and therapist together) |
| SE8b. If you answered NO, please do you think you would have liked to receive this/them? | ◯ No<br>◯ Don't know<br>◯ Yes<br>(If you do not meet family and therapist together) |

| | |
|---|---|
| SE9. In the last year, did you have a place in sheltered accommodation (e.g. foster home/family placement scheme, group home, hostel with staff available for help)? | ◯ Yes<br>◯ No<br>(Have a place in sheltered accommodation) |
| SE9a. If you answered YES, please what is your overall feeling about this/them? | ◯ Terrible<br>◯ Mostly dissatisfied<br>◯ Mixed<br>◯ Mostly satisfied<br>◯ Excellent<br>(If you have sheltered accommodation) |
| SE9b. If you answered NO, please do you think you would have liked to receive this/them? | ◯ No<br>◯ Don't know<br>◯ Yes<br>(If you have not received sheltered accommodation) |
| SE10. In the last year, did you have the opportunity to take part in leisure activities organized by the mental health services? | ◯ Yes<br>◯ No<br>(Have the opportunity to take part in leisure activities) |
| SE10a. If you answered YES, please what is your overall feeling about this/them? | ◯ Yes<br>◯ No<br>(If you have opportunity to receive leisure activities) |
| SE10b. If you answered NO, please do you think you would have liked to receive this/them? | ◯ No<br>◯ Don't know<br>◯ Yes<br>(If you have not received leisure activities) |
| SE11. In the last year, did you have group psychotherapy (e.g. meetings of a group of patients with one or more therapists with the aim of improving the patients understanding of their problems and/or changing their behaviour)? | ◯ Yes<br>◯ No<br>(Receive group psychotherapy) |
| SE11a. If you answered YES, please what is your overall feeling about this/them? | ◯ Terrible<br>◯ Mostly dissatisfied<br>◯ Mixed<br>◯ Mostly satisfied<br>◯ Excellent<br>(If you have received group psychotherapy) |
| SE11b. If you answered NO, do you think you would have liked to receive this/them? | ◯ No<br>◯ Don't know<br>◯ Yes<br>(If you have not received group psychotherapy) |
| SE12. In the last year, did you have any sheltered work? | ◯ Yes<br>◯ No<br>(Received sheltered work) |

| | |
|---|---|
| SE12a. If you answered YES, please what is your overall feeling about this/them? | ◯ Terrible<br>◯ Mostly dissatisfied<br>◯ Mixed<br>◯ Mostly satisfied<br>◯ Excellent<br>(If you have received a sheltered work) |
| SE12b. If you answered NO, please do you think you would have liked to receive this/them? | ◯ No<br>◯ Don't know<br>◯ Yes<br>(If you have not received sheltered work) |
| SE13. In the last year, did you have any voluntary admission to a psychiatric hospital? | ◯ Yes<br>◯ No<br>(Have a voluntary admission) |
| SE13a. If you answered YES, please what is your overall feeling about this/them? | ◯ Terrible<br>◯ Mostly dissatisfied<br>◯ Mixed<br>◯ Mostly satisfied<br>◯ Excellent<br>(If you have voluntary admission) |
| SE13b. If you answered NO, please do you think you would have liked to receive this/them? | ◯ No<br>◯ Don't know<br>◯ Yes<br>(If you have received voluntary admission) |
| SE14. In the last year, did you have practical help at home from the service (e.g. companionship, home help, etc.)? | ◯ Yes<br>◯ No<br>(Received practical health at home eg. companionship) |
| SE14a. If you answered YES, please what is your overall feeling about this/them? | ◯ Terrible<br>◯ Mostly dissatisfied<br>◯ Mixed<br>◯ Mostly satisfied<br>◯ Excellent<br>(If you have received practical help at home eg. companionship) |
| SE14b. If you answered NO,please do you think you would have liked to receive this/them? | ◯ No<br>◯ Don't know<br>◯ Yes<br>(If you have not received practical help at home eg. companionship) |
| SE15. In the last year, did you have help from the service obtaining welfare benefits or exemptions (e.g. Disability Allowance, Council Tax, etc.)? | ◯ Yes<br>◯ No<br>(If you received services in obtaining welfare or exemptions) |
| SE15a. If you answered YES, please what is your overall feeling about this/them? | ◯ Terrible<br>◯ Mostly dissatisfied<br>◯ Mixed<br>◯ Mostly satisfied<br>◯ Excellent<br>(If you received services in obtaining welfare benefits eg Disability Common Fund) |

| | |
|---|---|
| SE15b. If you answered NO, please do you think you would have liked to receive this/them? | ◯ No<br>◯ Don't know<br>◯ Yes<br>(If you have not received services in obtaining disability benefit) |
| SE16. In the last year, did you have help from the service finding open employment? | ◯ Yes<br>◯ No<br>(Help from services in finding open employment) |
| SE16a. If you answered YES, please what is your overall feeling about this/them? | ◯ Terrible<br>◯ Mostly dissatisfied<br>◯ Mixed<br>◯ Mostly satisfied<br>◯ Excellent<br>(If you received help from services in finding open employment) |
| SE16b. If you answered NO, please do you think you would have liked to receive this/them? | ◯ No<br>◯ Don't know<br>◯ Yes<br>(If you have not received services in finding open employment) |
| SE17. In the last year, did you receive help from the service to join in leisure activities separate from the mental health services (e.g. sports clubs, adult education, etc.)? | ◯ Yes<br>◯ No<br>(Received leisure activities separate from mental health services) |
| SE17a. If you answered YES, please what is your overall feeling about this/them? | ◯ Terrible<br>◯ Mostly dissatisfied<br>◯ Mixed<br>◯ Mostly satisfied<br>◯ Excellent<br>(If you have received leisure activities separate from mental health services) |
| SE17b. If you answered NO, please do you think you would have liked to receive this/them? | ◯ No<br>◯ Don't know<br>◯ Yes<br>(If you have not received leisure activities separate from mental health services) |

**In this section, I will be asking you questions about your overall satisfaction regarding the mental health services you have received in the last year. I will read out each question to you, along with the five response options (1= Terrible, 2 = Mostly dissatisfied, 3= Mixed, 4 Mostly satisfied and 5 Excellent). Please choose the answer that appears most appropriate. If you are unsure about which response to give to a question, the first response you think of is often the best one.**

| | Terrible | Mostly dissatisfied | Mixed | Mostly satisfied | Excellent |
|---|---|---|---|---|---|
| What is your overall feeling about the amount of help you have received? | ○ | ○ | ○ | ○ | ○ |
| What is your overall feeling about the kinds of service offered to you? | ○ | ○ | ○ | ○ | ○ |
| In an overall, general sense, what is your feeling about the service you have received? | ○ | ○ | ○ | ○ | ○ |

**In this section, we are going to talk about about the difficulties you experienced due to health conditions. The health conditions here include diseases or illnesses, other health problems that may be short or long lasting, injuries, mental or emotional problems, and problems with alcohol or drugs. Please choose the answer which is the best description of your experience over the past 30 days. I will read out each question to you, along with the five response options (1= None, 2 = Mild, 3= Moderate, 4 Severe and 5 cannot do at all). Please choose the answer that appears most appropriate. If you are unsure about which response to give to a question, the first response you think of is often the best one.**

**In the past 30 days, how much difficulty did you have in understanding and communicating:**

| | Cannot do at all | Severe | Moderate | Mild | None |
|---|---|---|---|---|---|
| D1.1 How much difficulty did you have concentrating on doing something for ten minutes? | ○ | ○ | ○ | ○ | ○ |
| D1.2 How much difficulty did you have remembering to do important things? | ○ | ○ | ○ | ○ | ○ |
| D1.3 How much difficulty did you have analysing and finding solutions to Problems in day-to-day life? | ○ | ○ | ○ | ○ | ○ |
| D1.4 How much difficulty did you have learning a new task, for example, learning How to get to a new place? | ○ | ○ | ○ | ○ | ○ |

| | | | | | |
|---|---|---|---|---|---|
| D1.5 How much difficulty did you have generally understanding what people say? | ◯ | ◯ | ◯ | ◯ | ◯ |
| D1.6 How much difficulty did you have starting and maintaining a conversation? | ◯ | ◯ | ◯ | ◯ | ◯ |

**Let continue to talk about the difficulty you have in getting around in the last 30 days. I will read out each question to you, along with the five response options (1= None, 2 = Mild, 3= Moderate, 4 Severe and 5 cannot do at all). Please choose the answer that appears most appropriate. If you are unsure about which response to give to a question, the first response you think of is often the best one.**

**In the past 30 days, how much difficulty did you have in getting around:**

| | Cannot do at all | Severe | Moderate | Mild | None |
|---|---|---|---|---|---|
| D2.1 How much difficulty did you have standing for long periods such as 30 Minutes? | ◯ | ◯ | ◯ | ◯ | ◯ |
| D2.2 How much difficulty did you have standing up from sitting down? | ◯ | ◯ | ◯ | ◯ | ◯ |
| D2.3 How much difficulty did you have moving around inside your home? | ◯ | ◯ | ◯ | ◯ | ◯ |
| D2.4 How much difficulty did you have getting out of your home? | ◯ | ◯ | ◯ | ◯ | ◯ |
| D2.5 How much difficulty did you have walking a long distance such as a kilometre [or equivalent]? | ◯ | ◯ | ◯ | ◯ | ◯ |

**Let talk about the difficulty you have in self-care. Please choose the answer which is the best description of your experience over the past 30 days. I will read out each question to you, along with the five response options (1= None, 2 = Mild, 3= Moderate, 4 Severe and 5 cannot do at all). Please choose the answer that appears most appropriate. If you are unsure about which response to give to a question, the first response you think of is often the best one.**

**In the past 30 days, how much difficulty did you have in self-care:**

| | Cannot do at all | Severe | Moderate | Mild | None |
|---|---|---|---|---|---|
| D3.1 How much difficulty did you have washing your whole body? | ○ | ○ | ○ | ○ | ○ |
| D3.2 How much difficulty did you have getting dressed? | ○ | ○ | ○ | ○ | ○ |
| D3.3 How much difficulty did you have eating? | ○ | ○ | ○ | ○ | ○ |
| D3.4 How much difficulty did you have staying by yourself for a few days? | ○ | ○ | ○ | ○ | ○ |

**Now let talk about the difficulty you have in getting along with people. Please choose the answer which is the best description of your experience over the past 30 days. I will read out each question to you, along with the five response options (1= None, 2 = Mild, 3= Moderate, 4 Severe and 5 cannot do at all). Please choose the answer that appears most appropriate. If you are unsure about which response to give to a question, the first response you think of is often the best one.**

**In the past 30 days, how much difficulty did you have in getting along with people:**

| | Cannot do at all | Severe | Moderate | Mild | None |
|---|---|---|---|---|---|
| D4.1 How much difficulty did you have dealing with people you do not know? | ○ | ○ | ○ | ○ | ○ |
| D4.2 How much difficulty did you have maintaining a friendship? | ○ | ○ | ○ | ○ | ○ |
| D4.3 How much difficulty did you have getting along with people who are close to You? | ○ | ○ | ○ | ○ | ○ |
| D4.4 How much difficulty did you have making new friends? | ○ | ○ | ○ | ○ | ○ |
| D4.5 How much difficulty did you have sexual activities? | ○ | ○ | ○ | ○ | ○ |

**Let talk about the difficulty you have in life activities. Please choose the answer which is the best description of your experience over the past 30 days. I will read out each question to you, along with the five response options (1= None, 2 = Mild, 3= Moderate, 4 Severe and 5 cannot do at all) Please choose the answer that appears most appropriate. If you are unsure about which response to give to a question, the first response you think of is often the best one.**

**In the past 30 days, how much difficulty did you have in Life activities:**

| | Cannot do at all | Severe | Moderate | Mild | None |
|---|---|---|---|---|---|
| D5.1 How much difficulty did you have taking care of your household responsibilities? | ○ | ○ | ○ | ○ | ○ |
| D5.2 How much difficulty did you have doing most important household tasks Well? | ○ | ○ | ○ | ○ | ○ |
| D5.3 How much difficulty did you have in getting all the household work that you needed to do done? | ○ | ○ | ○ | ○ | ○ |
| D5.4 How much difficulty did you have getting your household work done as Quickly as needed? | ○ | ○ | ○ | ○ | ○ |
| D5.5 How much difficulty did you have your day-to-day work/school activity? | ○ | ○ | ○ | ○ | ○ |
| D5.6 How much difficulty did you have doing your most important work/school tasks well? | ○ | ○ | ○ | ○ | ○ |
| D5.7 How much difficulty did you have getting all the work done that you need to do? | ○ | ○ | ○ | ○ | ○ |
| D5.8 Getting your work done as quickly as needed? | ○ | ○ | ○ | ○ | ○ |

**Let talk about the difficulty you have in participating in society. Please choose the answer which is the best description of your experience over the past 30 days. I will read out each question to you, along with the five response options (1= None, 2 = Mild, 3= Moderate, 4 Severe and 5 cannot do at all) Please choose the answer that appears most appropriate. If you are unsure about which response to give to a question, the first response you think of is often the best one.**

**In the past 30 days, how much difficulty did you have in participation in society:**

| | Cannot do at all | Severe | Moderate | Mild | None |
|---|---|---|---|---|---|
| D6.1 How much of a problem did you have in joining in community activities (for example, festivities, religious or other activities) in the same way as anyone else can? | ○ | ○ | ○ | ○ | ○ |
| D6.2 How much of a problem did you have because of barriers or hindrances in the world around you? | ○ | ○ | ○ | ○ | ○ |
| D6.3 How much of a problem did you have living with dignity because of the attitudes and Actions of others? | ○ | ○ | ○ | ○ | ○ |
| D6.4 How much of a problem did you spend time on your health condition, or its consequences? | ○ | ○ | ○ | ○ | ○ |
| D6.5 How much have you been emotionally affected by your health condition? | ○ | ○ | ○ | ○ | ○ |
| D6.6 How much has your health been a drain on the financial resources of you or your family? | ○ | ○ | ○ | ○ | ○ |
| D6.7 How much of a problem did your family have because of your health problems? | ○ | ○ | ○ | ○ | ○ |
| D6.8 How much of a problem did you have in doing things by yourself for relaxation or pleasure? | ○ | ○ | ○ | ○ | ○ |

Overall, in the past 30 days, how many days were these difficulties present?

__________________________
(Number of days difficulties were present)

In the past 30 days, for how many days were you totally unable to carry out your usual activities or work because of any health condition?

__________________________
(Number of days unable to carry out usual activities)

In the past 30 days, not counting the days that you were totally unable, for how many days did you cut back or reduce your usual activities or work because of any health condition?

__________________________________
(Number of days you cut back or reduce your usual activities)

What is the type of mental condition of the consumer?

- ☐ Depression
- ☐ Schizophrenia
- ☐ Bipolar Affected Disorder
- ☐ Suicide Attempt
- ☐ Substance induced (Cannabis Abuse)
- ☐ Psychotic symptoms
- ☐ Opioid Dependence
- ☐ Other

(Type of mental condition)

If you answered other, please state?

__________________________________________
(Other mental condition)

What is the stage of the mental condition?

__________________________________
(Mental Health Examination Status)
